# PILOT RESULTS OF A GAMIFIED TRAINING FOR CERTIFIED NURSING ASSISTANTS ON AGE-FRIENDLY HEALTHCARE

**DOI:** 10.1093/geroni/igae098.4047

**Published:** 2024-12-31

**Authors:** David Wihry, Susan Wehry, Angela Hunt, Judith Metcalf, Victoria Spofford

**Affiliations:** University of Maine, South Portland, Maine, United States; University of New England, Portland, Maine, United States; The Cedars, Portland, Maine, United States; University of New England, Biddeford, Maine, United States; University of New England, Biddeford, Maine, United States

## Abstract

Certified Nursing Assistants (CNAs) are an integral part of supporting the health and wellbeing of older adults within long-term care (LTC) settings. As the Age-Friendly Healthcare movement expands to include LTC, it will be important to ensure effective and accessible training for CNAs on Age-Friendly Healthcare competencies. This poster will present the results of a pilot study examining the usability and satisfaction with an app-based training on the 4Ms of Age-Friendly Healthcare (What Matters, Medication, Mentation, Mobility). The modules are tailored to the practice needs of CNAs and use principles of gaming to increase uptake of the training. A post training survey was distributed to 127 CNAs from eight LTC communities who completed the curriculum, with a 79% response rate. 78% agreed they would prefer to learn this way versus alternative learning methods such as online learning, classroom learning, etc. (12% neither agreed nor disagreed, 10% disagreed). 85% agreed that they believe gamification is a successful delivery method for learning (8% neither agreed nor disagreed, 7% disagreed). 80% agreed that they would recommend the training program to their co-workers (13% neither agreed nor disagreed, 7% disagreed). The number of participants stating that they had a “very good” level of knowledge of Age-Friendly Healthcare increased from 25% to 39% using a retrospective pre-post rating. Qualitative feedback highlighted interactivity, brevity, and ability to engage the individual as key themes about the value of the training modules. Implications for use of gamification in Age-Friendly Healthcare training will be presented.

